# Classification of Blood Pressure Levels Based on Photoplethysmogram and Electrocardiogram Signals with a Concatenated Convolutional Neural Network

**DOI:** 10.3390/diagnostics12112886

**Published:** 2022-11-21

**Authors:** Yunendah Nur Fuadah, Ki Moo Lim

**Affiliations:** 1 Computational Medicine Lab, Department of IT Convergence Engineering, Kumoh National Institute of Technology, Gumi 39177, Republic of Korea; 2 School of Electrical Engineering, Telkom University, Bandung 40257, Indonesia; 3 Computational Medicine Lab, Department of Medical IT Convergence Engineering, Kumoh National Institute of Technology, Gumi 39177, Republic of Korea

**Keywords:** blood pressure levels, hypertension, PPG signal, ECG signal, convolutional neural network

## Abstract

Hypertension is a severe public health issue worldwide that significantly increases the risk of cardiac vascular disease, stroke, brain hemorrhage, and renal dysfunction. Early screening of blood pressure (BP) levels is essential to prevent the dangerous complication associated with hypertension as the leading cause of death. Recent studies have focused on employing photoplethysmograms (PPG) with machine learning to classify BP levels. However, several studies claimed that electrocardiograms (ECG) also strongly correlate with blood pressure. Therefore, we proposed a concatenated convolutional neural network which integrated the features extracted from PPG and ECG signals. This study used the MIMIC III dataset, which provided PPG, ECG, and arterial blood pressure (ABP) signals. A total of 14,298 signal segments were obtained from 221 patients, which were divided into 9150 signals of train data, 2288 signals of validation data, and 2860 signals of test data. In the training process, five-fold cross-validation was applied to select the best model with the highest classification performance. The proposed concatenated CNN architecture using PPG and ECG obtained the highest test accuracy of 94.56–95.15% with a 95% confidence interval in classifying BP levels into hypotension, normotension, prehypertension, hypertension stage 1, and hypertension stage 2. The result shows that the proposed method is a promising solution to categorize BP levels effectively, assisting medical personnel in making a clinical diagnosis.

## 1. Introduction

Hypertension is the most significant reversible risk factor for cardiovascular heart disease, stroke, brain hemorrhage, chronic kidney, and other severe illnesses, which are responsible for causing 8.5 million mortalities worldwide [1,2,3]. According to the American heart society report, 1.56 billion people globally will suffer from hypertension by 2025, with more than half living in developing countries with insufficient healthcare services to manage hypertension [4]. The high prevalence of hypertension as a silent killer is due to hypertension not showing symptoms in the early stages, only emerging after the severe stage has been reached [5]. Hypertension occurs when the blood pressure (BP) in the arteries rises and forces the heart to pump harder to deliver oxygenated blood to other parts of the body [6]. Therefore, screening of blood pressure levels is essential for early diagnosis of hypertension to prevent severe complications and to provide the proper medical treatment for the patient [7].

A sphygmomanometer (cuff-based monitoring) is frequently used to measure blood pressure and is essential in monitoring hypertension [8]. However, cuff-based monitoring causes artery compression and requires the proper procedures while measuring BP [9]. Furthermore, if the user misuses a cuff-based device, an incorrect diagnosis will be obtained. As a result, a new cuff-less BP screening and hypertension detection method is being developed [10,11,12,13].

Photoplethysmography waveform is commonly used to develop a cuff-less methodology for BP prediction [14,15,16,17] or high BP risk categorization. The acquisition process of PPG can operate in transmission or reflection modes depending on the transmitter (light source) and receiver (photodetector) position. The transmission mode arranges the light source and photodetector position opposite each other, with layers of skin tissues in between [18]. The photodetector detects the residual light from the source after the tissue has absorbed it. Typically, this PPG sensor is mainly employed for measuring PPG at the distal body area, including fingers, toes, and earlobes. The finger versions are extensively utilized in medical applications such as pulse oximeters. Meanwhile, the earlobe version is frequently utilized for vitality monitoring [19].

Furthermore, the photodetector is positioned next to the light source for the PPG sensor that operates based on the reflecting principle. The photodetector detects the reflected light on the same body surface. Therefore, the reflecting principle provides higher flexibility for measuring PPG signals in different body areas, such as the wrist, forehead, esophagus, and carotid, where the transmission principle cannot measure well. As a result, they are ideal to be implemented in noninvasive wearable technologies for long-term monitoring.

The fundamental hardware components of the PPG measurement system are a light emitting diode (LED) and a photosensitive diode that extract the PPG signal. The LED emits light red or infrared to illuminate the skin on the wrist, fingertip, earlobe, or forehead [20]. Meanwhile, the photosensitive diode monitors the tissue’s shifting light absorption over time and can detect variations in blood volume. The PPG signal records the signal produced by blood flow fluctuation in the blood vessels. Variations in heart rate cause variations in intravascular blood flow (per unit). The inductive voltage recorded by light sensors varies in response to fluctuations in blood flow. During systole, light sensors absorb the maximum light. The PPG signal amplitude is proportional to the tissue’s blood flow and outflow variation. Therefore, the PPG signal provides physiological information related to the cardiovascular circulation system, systolic and diastolic heart activity, the peripheral microcirculation system’s network, and hemorheological and hemodynamic data [21].

Several studies have observed that PPG waveforms correlate with cardiovascular pathology [22]. Pulse rate variability (PRV), which is measured from peak to peak of PPG signals, is considered a surrogate method for heart rate variability assessed from ECG signals under resting conditions [23]. A study proposed by Mejía et al. analyzed the reliable duration of the PPG segment to extract pulse rate variability (PRV) from PPG signals [24]. Their study reported that 120 s PPG segment duration could be used to determine PRV accurately. However, PRV and HRV could differ under dynamic circumstances, such as exercise and mental stress [25].

In addition, researchers are interested in the arterial wave propagation theory based on ECG and PPG signals for estimating BP. The theory generates pulse arrival time (PAT), pulse transit time (PTT), and pulse wave velocity (PWV) as robust surrogate features to measure the conditions of the heart’s physiological systems, including blood pressure, arterial stiffness, and arterial compliance [26].

The other potential factors which attract researchers are the derivative features of PPG signals. Takazawa et al. claimed that PPG’s first and second derivatives are useful for representing spatiotemporal variations in PPG, including peak position, inflection point, number of peaks, and ascending and descending slope [27]. These features can be used as a substitute technique to find dicrotic and diastolic peaks that are challenging to determine in the original PPG waveforms. Liu et al. extracted 35 features from the PPG signal and its second derivative for BP estimation using support vector regression [28].

Meanwhile, Gupta et al. proposed PPG’s holistic nonlinear dynamics and moving slope features extracted from the third and fourth derivatives of PPG signals. They performed optimization of several machine learning algorithms, including random forest (RF), extreme gradient boosting (XGBoost), and support vector regression (SVR) to estimate the BP values. Their study successfully obtained Grade A on the British Hypertension Society (BHS) standard, which confirmed the robustness of the proposed study to estimate BP [29].

Previous studies used handcrafted features from the PPG as input to the traditional machine learning algorithm, which potentially does not represent the whole physiological characteristics of PPG signals. Therefore, Khodabakhshi et al. used the whole PPG sequence and nonlinear handcrafted features as input to the parallel convolutional neural network [30]. The combination of the chaotic features and the machine-learned features from PPG signals can be effectively applied to improve the performance of monitoring BP.

In recent studies, several researchers used machine learning with PPG features to automatically predict or categorize blood pressure levels. Wu et al. proposed continuous wavelet transforms to transform PPG signals into 224 × 224 × 3 scalogram images [31]. The scalogram images of the PPG signals are be used as input to the 2D CNN, which consists of two convolutional layers followed by pooling layers in the feature extraction layers and two hidden layers in the classification layers. Their study reported the highest classification accuracy of 90% in classifying normal and abnormal conditions of blood pressure levels.

Moreover, Sun et al. proposed a 2D convolutional neural network based on the Hilbert Huang Transform (HHT) method using a 10 s segment of PPG signal [32]. They considered the first and second derivatives of the PPG signal related to atherosclerosis and vascular elasticity. Furthermore, the PPG signal and its derivative were transformed into 224 × 224 × 3 spectrogram images using the HHT method and used as input to the Alexnet architecture. Their study successfully obtained a classification accuracy of 98.90% in classifying normotension vs. hypertension, an accuracy of 85.80% in classifying normotension vs. prehypertension, and 93.54% in classifying normotension vs. prehypertension vs. hypertension.

Yen et al. used the PPG signal as input to the deep residual network convolutional neural network (ResNetCNN) and bidirectional long short-term memory (BILSTM) [33]. Their study reported a classification accuracy of 76% in classifying hypertension into four classes: normotension, prehypertension, hypertension stage 1, and hypertension stage 2. Their proposed method expanded the classification of blood pressure levels into four classes: normotension, prehypertension, hypertension stage 1, and hypertension stage 2. However, the model is still overfitting and only performs well on the training set by obtaining a training accuracy of 100% but still lacks generalization ability for the unseen dataset by achieving a test accuracy of 76%.

While the previous studies focused on employing the PPG signal to categorize blood pressure levels, several researchers reported that electrocardiogram (ECG) signals strongly correlate with arterial blood pressure [34]. ECG signals represent the heart’s electrical activity [35,36]. The electrical impulse causes the rhythmic contraction of the heart [35,36,37]. Blood is discharged from the atrium and ventricle during heart contraction or systole. Meanwhile, the diastole or relaxation condition is when the atrium and ventricle fill with blood. Regarding ECG signal morphology, systole conditions can be identified from the R peak wave to the T peak wave. In contrast, diastole conditions can be identified from the T peak wave to the R peak wave [38]. Hypertension causes anatomical and functional changes in the heart, which leads to left ventricular hypertrophy (LVH), diastolic dysfunction, and heart failure [39]. Meanwhile, several studies showed that ECG also correlated with hypotension conditions. Yeon Jo et al. reported an AUC score of 0.957 (0.954–0.960) using ABP, ECG, and electroencephalogram (EEG) signals as input to deep learning models using ResNet architecture, which contains 12 residual blocks and one linear layer. [40] Moreover, Bae et al. used heart rate information corresponding to time series data and patient baseline information as input to a multilayer perceptron [41]. Their study aim was to validate heart rate difference and heart rate slope features correlated with hypotension conditions. Their findings reported the highest accuracy of 81.5% in predicting hypotension conditions.

Soh et al. proposed a deep-learning model coupled with an ECG signal to detect hypertension [42]. They developed four convolutional layers followed by a max pooling layer with fully connected layers. Their study reported a high classification accuracy of 99.99% in classifying normotension and hypertension of ECG signals. Jain et al. proposed a two-stage deep CNN architecture to classify low-risk and high-risk hypertension based on ECG signals [43]. The first stage configuration of the CNN model consists of two convolution layers followed by two pooling layers with two fully connected layers. Meanwhile, the second stage configuration of CNN consists of four convolution layers followed by four pooling layers with three fully connected layers. The first and second stages of the CNN model obtained classification accuracy of 96.68% and 90.08%, respectively. Rajput et al. proposed an optimal wavelet filter bank to classify low-risk and high-risk hypertension. Their study reported a classification accuracy of 99.95% [44]. Sharma et al. reported 98.05% test accuracy in classifying normotension and hypertension using a support vector machine (SVM) classifier [45].

Instead of using physiological information from single-wave PPG or ECG, several studies extracted the features from PPG and ECG. Liang et al. used arterial propagation theory, which generated features from PPG and ECG signals [46]. Furthermore, these features were classified using several classifier models, including logistic regression, AdaBoost tree, bagged tree, and k-nearest neighbor (K-NN). The highest classification performance was obtained using K-NN by obtaining the F1-scores of 84.34% in classifying normotension and prehypertension, 94.84% for classifying normotension versus hypertension, and 88.49% in classifying three classes including normotension, prehypertension, and hypertension. The simultaneous collection of PPG and ECG signals provided good performance in detecting hypertension [26]. However, the study proposed by Liang et al. still used handcrafted features and traditional machine learning. Kuzmanov et al. proposed a CNN and long short-term memory networks (LSTM) model with PPG and ECG signals. Their study reported an AUC score of 0.74 in classifying two conditions of blood pressure, including hypotension and not hypotension [47]. In addition, Kuzmanov et al. reported an AUC score of 0.76 in classifying normotension, prehypertension, and hypertension using CNN and the gated recurrent unit (GRU) model with PPG and ECG [48]. According to the results, the performance still needs to be improved by expanding the dataset and trying different models.

The aforementioned studies showed that PPG and ECG are heavily reliant on blood pressure and can potentially be used to employ the classification model of BP levels. Moreover, the classification performance in classifying BP levels obtained high accuracy, especially for binary classification. However, the performance needs to be improved for the multi-classification of BP levels. Therefore, this study proposes a concatenated CNN model using PPG and ECG signals as inputs to the classifier model. In order to assess the suitable input and evaluate the performance of the concatenated CNN, we compare it with a single 1D-CNN using PPG or ECG signals only. The main contributions of this study are providing a parallel convolutional layer to extract the critical features from PPG and ECG signals and combining the physiological information of these signals using concatenated CNN to generate detailed features representing the characteristics of each blood pressure level. These findings provide a promising solution to improve the classification performance of blood pressure levels into five categories: hypotension, normotension, prehypertension, hypertension stage 1, and hypertension stage 2.

## 2. Materials and Methods

### 2.1. Dataset and Preprocessing

**Figure 1:**
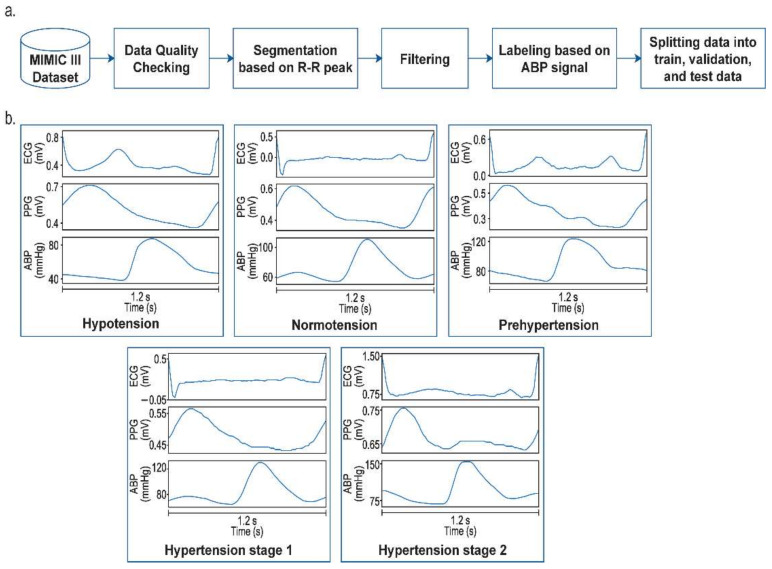
(**a**) The proposed preprocessing steps. (**b**) Preprocessing results.

The PPG, ECG, and ABP signals of 221 patients were selected from Multiparameter Intelligent Monitoring in Intensive Care (MIMIC III) dataset, which can be accessed on the Physionet website [49]. The dataset was collected from patients in the Critical Unit of Beth Israel Deaconess Medical Center. Preprocessing consisted of several steps, as shown in Figure 1. At first, we handled some missing data and checked the signal quality that was used as a dataset for further processing. The segmentation process was performed based on R-R peak detection of the ECG signal to generate one cycle of PPG and ABP signals.

Furthermore, a bandpass filter (BPF) with cut-offs at 0.5–10 Hz and 0.5–40 Hz was applied to remove noises in PPG and ECG signals, respectively [26]. Since the length of PPG and ECG segment signals varies, we interpolated the signals to possess the same sizes. Therefore, all signals had the same number of samples (150 samples) as input to the CNN model. The label for each segment was assigned based on the systole and diastole value extracted from ABP signals. The American Heart Association standard divides blood pressure values into several categories: hypotension, normotension, prehypertension, hypertension stage 1, and hypertension stage 2, as shown in Table 1. A total number of 14,298 signal segments were generated and divided into 9150 signals of train data, 2288 signals of validation data, and 2860 signals of test data.

### 2.2. Concatenated 1D CNN Architecture

**Figure 2:**
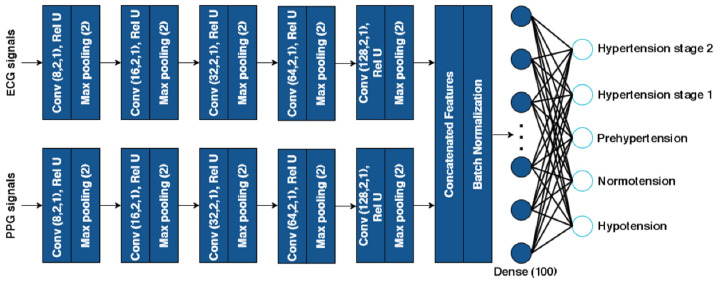
The proposed concatenated 1D CNN architecture.

This study used PPG and ECG signals as input to the concatenated 1-D CNN architectures, as shown in Figure 2. In the feature extraction layer, the computing output map of one layer becomes the input of the subsequent layer during forwarding propagation. This input is then convolved with specific kernels, as shown in Equation (1) [50].

(1) $$x_{k}^{h}=\overset{N_{h-1}}{\underset{i=1}{\sum }}conv1D\;(w_{ik}^{h-1}.\;s_{i}^{h-1})+b_{k}^{h},$$

where $x_{k}^{h}$ is the input of layer h, and $w_{ik}^{h-1}$ and $s_{i}^{h-1}$ stand for the kernel and output of the $i^{th}$ neuron at layer h−1, respectively. Meanwhile, $\;b_{k}^{h}$ is the bias of the $k^{th}$ neuron at layer h. Furthermore, the activation function computes the output $y_{k}^{h}\;$of the input $x_{k}^{h}$ for each neuron in a middle layer, as shown in Equation (2) [49].

(2) $$y_{k}^{h}=f\left(x_{k}^{h}\right)\;\mathrm{and}\;s_{k}^{h}=y_{k}^{h}\;↓ss,$$

where $\;s_{k}^{h}$ is the output of the $k^{th}$ neuron of layer h, and ↓ss is the downsampling operation of scalar factor (ss).

This study designed CNN architectures with different convolutional depth layers. The CNN architectures consist of one convolutional layer with 8 filters; two convolutional layers with 8 and 16 filters; three convolutional layers with 8, 16, and 32 filters; four convolutional layers with 8, 16, 32, and 64 filters; five convolutional layers, with the number of filters from layers one through five being 8, 16, 32, 64, and 128, respectively. We applied a small kernel size with stride one and applied the rectified linear unit activation function (Rel-U) in the convolutional layer to extract the essential characteristics of ECG and PPG signals in more detail. Furthermore, max pooling was applied following each convolutional layer to downsample the features.

The PPG and ECG signal feature maps were concatenated as input to the fully connected classification layer. The formulation of concatenation fusion is shown in Equation (3), which concatenates the two inputs of the extracted feature maps from PPG and ECG signals at the same location, as shown in Equation (4) [50].

(3) $$y^{cat}=f^{cat}\left(x^{a},\;x^{b}\right),$$

(4) $$y_{i.j.1:n}^{sum}=X_{i.j.n}^{a},\;y_{i.j.n+1:2n}^{sum}=X_{i.j.n}^{b},$$

where $1\leq i\leq H,\;1\leq j\leq W,\;1\leq n\leq N,\;\mathrm{and}\;y\epsilon \;R^{H\times W\times 2N}$.

$x^{a}\;\mathrm{and}\;x^{b}$ are the fusion function input, and y is the output. Meanwhile, H, W, and N stand for height, weight, and the number of channels of the feature maps, respectively. After concatenating the feature maps, we applied batch normalization, a hidden layer, and the output layer with a SoftMax activation function, which was classified into five groups: hypotension, normotension, prehypertension, hypertension stage 1, and hypertension stage 2.

In order to assess deep neural networks for generalization ability in classifying the unseen data, we applied 5-fold cross-validation. As for the regularization technique, we applied callback to monitor the particular metric, including validation accuracy and loss. The model checkpoint automatically saved the network weight when the loss in validation decreased. We used Adam as an optimizer method and categorical cross-entropy as a loss function to reduce error during training with a learning rate of 0.001.

### 2.3. Evaluation Matrix

We used a confusion matrix to measure the system performance, including accuracy precision, recall, and f1-score, as shown in Equations (5)–(8), respectively [51]. A result is considered true positive (*TP*) if the model accurately predicts the positive class. Contrarily, a true negative (*TN*) is a result in which the model accurately predicts the negative class. A false positive (*FP*) results when negative data is incorrectly categorized as positive. False negative (*FN*) results, on the other hand, are when positive data is incorrectly categorized as negative.

(5) $$Accuracy=\frac{\left(TP+TN\right)}{\left(TP+FP+TN+FN\right)},$$

(6) $$Precision=\frac{\left(TP\right)}{\left(TP+FP\right)},$$

(7) $$Recall=\frac{\left(TP\right)}{\left(TP+FN\right)},$$

(8) $$F1\;score=2\cdot \frac{Recall\;\cdot \;Precision}{Recall+Precision}.$$

## 3. Results

A total of 2860 test data signals were employed, including 407 data on hypotension, 1200 data on normotension, 182 data on prehypertension, 540 data on hypertension stage 1, and 531 data on hypertension stage 2. We used test data signals to validate the models produced by the training process using five-fold cross-validation. Table 2 presents the classification accuracy results with a 95% confidence interval that used the PPG signal, ECG signal, or both PPG and ECG signals as inputs to the CNN architecture.

As shown in Table 1, the PPG signal as input to the 1D CNN architecture with five convolutional layers obtained the highest classification test accuracy of 57.97–61.23%. Furthermore, the highest classification test accuracy for the ECG signal as input is 75.63–79.57%. The performance significantly improved using PPG and ECG signals as input to the concatenated CNN compared to using PPG or ECG only as input to the CNN architecture. Moreover, we used the ANOVA test for statistical analysis with statistical significance at p<0.05. The features combined of PPG and ECG showed the most statistical significance with a p-value of 4.72 $\times 10^{-12}$ in differentiating five blood pressure categories. Therefore, PPG and ECG signals as input to the proposed concatenated CNN architecture with five convolutional layers provided the highest classification accuracy compared to the single wave of PPG or ECG signals as input to the CNN architecture. The highest performance obtained was 100% for the training and validation data. Meanwhile, the performance for the test data achieved classification accuracy of 94.56–95.15% with 95% confidence intervals after evaluation using five selected models generated by five-fold cross-validation. There was a difference in accuracy performance between training and testing accuracy due to the imbalanced dataset.

For further analysis using PPG and ECG signals as input, we tested five architectures based on the number of filters and the depth of the convolutional layer to determine the optimal configuration of concatenated CNN. Architectures 1–5 consisted of 1, 2, 3, 4, and 5 convolutional layers, respectively, for both PPG and ECG, which were designed in parallel. Table 3 presents the detailed performance for classifying each blood pressure level using five architectures. The best architecture was selected based on the performance of precision, recall, F1 score, and AUC score after evaluation using models generated by five-fold cross-validation. As shown in Table 3, architecture 5 achieved the highest results of test data compared with the other architectures by obtaining 0.93–0.95 of precision, recall, and F1-score, respectively with an AUC score of 0.98–0.99. Architecture 5 was followed by architecture 4, 3, and 2, which obtained a similar performance. Meanwhile, architecture 1, consisting of 1 convolutional layer, obtained the lowest performance.

The results showed that the number of convolutional layers substantially affects the classification performance of the feature maps generated by the feature extraction layers. The appropriate number of convolutional layers was determined by simulating and evaluating the system’s classification performance. If the model is too simplistic, it cannot extract the unique characteristics. In contrast, a deep model will increase model complexity and computational time.

**Figure 3:**
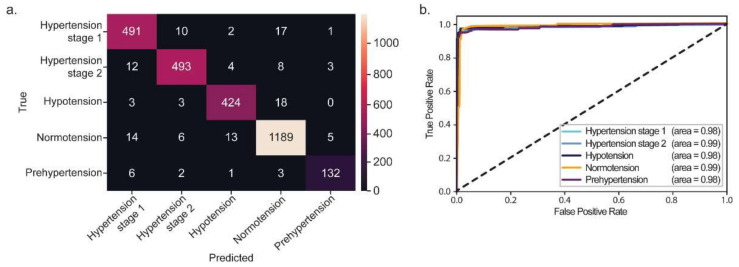
The performance results of test data. (**a**) Confusion matrix. (**b**) AUC score.

The confusion matrix of architecture 5 showed that the model successfully classified these blood pressure levels of test data as mostly accurate according to their class, as shown in Figure 3a. The receiver operating characteristics (ROC) curve consisted of the *y*-axis as true positive rates (TPR) and the *x*-axis as the false positive rates (FPR), as shown in Figure 3b. The area under curve (AUC) score showed the classifier’s ability to classify between classes. The model’s ability to differentiate between blood level categories of test data improved with the AUC scores of 0.98–0.99. Therefore, we can conclude that the suggested model effectively generalizes the test datasets.

## 4. Discussion

Physiological information from PPG and ECG signals is used by researchers to develop computer-aided diagnostic tools that can automatically classify blood pressure levels. Table 4 presents several previous studies related to hypertension classification, which used features from a single wave of PPG or ECG signals and used both signals as input to machine learning and deep learning models. Several advantages and disadvantages of each model proposed in the previous studies related to blood pressure level classification will be discussed in this section.

The studies which transformed the PPG signal into 224 × 224 × 3 2D scalogram images, such as the study proposed by Wu et al. and Sun et al., obtained an accuracy of 90% in classifying blood pressure into two categories and an accuracy of 93.54% in classifying three categories of blood pressure levels, respectively, using a 2D CNN model. Wu et al. transformed the PPG signals using continuous wavelet transforms into scalogram images and used a simple 2D CNN model using two convolutional layers [31]. Meanwhile, Sun et al. transformed the first derivative of the PPG signal into a scalogram image using the the Hilbert Huang transform and used Alexnet architecture, which consists of eight layers [32]. The results show that a higher number of layers extract the features in more detail and can improve the classification performance. However, converting the PPG signal into 2D images requires more computational time than directly using the PPG signal’s time series data.

The study proposed by Yen et al. used PPG signal time series data as input to the BILSTM and ResNet [33]. Moreover, they expanded the classification of blood pressure levels into four categories: normotension, prehypertension, hypertension stage 1, and hypertension stage 2. However, the performance indicated overfitting by providing 100% accuracy for train data and 76% for test data. There is a huge difference between the train and test accuracy due to the imbalanced dataset of four blood pressure levels.

In addition, several researchers approved the accuracy improvement for blood pressure classification, which extracted physiological information from ECG signals. The study proposed by Soh et al. used the time series data of ECG signals as input to the CNN model, which consists of four convolutional layers followed by a max pooling layer [42]. In evaluating the model performance, they applied 10-fold cross-validation (CV) and left out one validation technique. Their study reported 99.99% accuracy in classifying normotension and hypertension for both validation techniques. The results showed that the proposed model has high robustness in diagnosing hypertension.

A study proposed by Rajput et al. used a two-stage deep CNN architecture to classify low-risk and high-risk hypertension using multi-lead ECG signals [44]. Their study claimed that features extracted from ECG signals are clinically significant at *p* < 0.001. Moreover, the study achieved a robust model after evaluation using three types of cross-validation models, including random hold-out cross CV, five-fold CV, and LOOCV. The first stage of deep CNN, which consists of two convolutional layers, two pooling layers, and two fully connected layers, obtained an accuracy of 99.68% in detecting hypertension. Furthermore, the second stage of deep CNN consists of four convolutional layers, four pooling layers, and three-fully connected layers categorized low-risk and high-risk hypertension conditions with 90.98% accuracy. Their study’s limitation is only considered three-lead ECG signals from 221 subjects. Therefore, for future works, they will expand the number of channels from more subjects and extend for classification of prehypertension, moderate hypertension, and severe hypertension classes.

Sharma et al. evaluated 23 studies related to hypertension detection using physiological signals, including ECG, HRV, PPG, and ballistocardiograph signals (BCG) [45]. According to their findings, ECG signals and HRV features provided the best performance compared with the other features. They tested several supervised machine learning algorithms, including ensemble begged tress (EBT), k-nearest neighbor (K-NN), and support vector machine (SVM). The highest classification performance obtained 98.05% accuracy with an AUC score of 1. For further research, deep learning architecture with a large database will be used to classify normotension, high-risk, and low-risk hypertension.

The result showed that physiological information from PPG and ECG signals strongly correlates with blood pressure measurement. Therefore, several studies used PPG and ECG signals to classify blood pressure levels. A study proposed by Liang et al. provided an F1-score of 88.49% in classifying normotension, prehypertension, and hypertension [46]. However, there are several limitations due to the handcrafted features extracted not representing the critical information of the whole signals and still using traditional machine learning algorithms to classify the features. Furthermore, a study proposed by Kuzmanov et al. extracted the features directly from the PPG and ECG with a duration of 8 s using a deep learning model. The proposed study provided 76% accuracy using LSTM and CNN in classifying hypotension and not hypotension [47]. Furthermore, Kuzmanov et al. extended the classification system for three blood pressure level categories and obtained 78% accuracy using GRU and CNN models [48]. According to the results, the performance is still affected by false detection and needs to be improved. For further research, they will adjust the segment size for all datasets and develop regression models for BP estimation using Big Data with a deep learning approach.

To address several limitations in previous studies, which still used handcrafted features and traditional machine learning algorithms, This study extracted the features directly from time series data of PPG and ECG signals and combined the features using a concatenated CNN model. Furthermore, the classification system extends to classify blood pressure levels into five categories based on the American Heart Society standard: hypotension, normotension, prehypertension, hypertension stage 1, and hypertension stage 2. Based on the ANOVA test, features from PPG and ECG signals show the most statistical significance with the *p*-value of 4.72 $\times 10^{-12}$ (*p* < 0.05) compared to a single wave of PPG or ECG signals.

In designing the configuration of the CNN architecture, the depth of layers and the number of filters should be considered. These variables affect both model complexity and feature maps. The model cannot extract the significant features if the configuration is too simple. On the other side, if the model is too deep, the computational time for training will be prolonged and increase the model’s complexity. As a result, we assessed five configurations using varied convolutional layer depths. As shown in Table 2, the CNN architecture which consists of five convolutional layers showed the highest performance in classifying blood pressure levels by obtaining 100% training accuracy, 100% validation accuracy, and 94.56–95.15% test accuracy with a 95% confidence interval. There was a 5% difference between training and testing, which indicated that it was still affected by false detection. However, the proposed model showed a promising solution in classifying five categories of BP levels. As a novel approach, our proposed concatenated CNN architecture uses PPG and ECG as input to the model.

Indeed, the five convolutional layers with 8, 16, 32, 64, and 128 filters for layers one through five, respectively, using a small filter size for each layer, produced feature maps by extracting the physiological information from PPG and ECG signals. The number of filters, which gradually increased with small filter size, extracted the comprehensive features and started to identify the low features that combine to generate more complex features. The concatenated layers fused previous models’ feature maps in more detail as input to the fully connected layers. Therefore, the proposed concatenated CNN architecture outperformed the previous studies that used PPG and ECG. Moreover, this study successfully expanded the classification system’s ability to classify five blood pressure levels.

Cuff-based monitoring is not a suitable option for long-term blood pressure monitoring due to inconvenience associated with blood pressure measurements. Meanwhile, catheterization is required for invasive blood pressure measurement, which is not commonly used for ambulatory disorders. Therefore, computer-aided diagnostic tools for monitoring and classifying blood pressure levels were developed using a deep neural network to extract the physiological characteristic from PPG and ECG signals. The performance of our proposed method showed a promising solution to be used as an assisting tool in diagnosing blood pressure levels. However, our study had several limitations: first, due to the imbalanced dataset, our result is affected by false detection. As a solution, for further research, we must expand the number of datasets. Second, our study required a clinical experiment with primary datasets that included comprehensive medical information related to diagnosing blood pressure levels, such as age, BMI, and medical history, to approach clinical viability.

## 5. Conclusions

This paper discussed the diagnostic system for blood pressure level classification based on physiological information extracted from PPG and ECG signals using concatenated CNN. By analyzing the statistical significance of each signal, the features from PPG and ECG were associated with the blood pressure levels by obtaining a higher classification accuracy compared with the single wave of PPG or ECG as input to the CNN model. Furthermore, five architectures of CNN models based on the depth of layers were observed to determine the optimal configuration of the proposed model. The concatenated CNN models, which consist of five convolutional layers, provided the highest performance in classifying blood pressure levels. The proposed model successfully extracted the PPG and ECG features directly and combined these signals’ essential information. Moreover, this study expanded the classification problem of blood levels from binary classification (normotension and hypertension) into five categories: hypotension, normotension, prehypertension, hypertension stage 1, and hypertension stage 2. According to the results, the system can be used as an additional tool to diagnose blood pressure levels. However, the proposed model still needs to be validated with a large number of data to confirm our proposed model’s clinical feasibility.

## Figures and Tables

**Table 1:** The American Heart Association standard for blood pressure levels.

BP Levels	Systolic (mmHg)		Diastolic (mmHg)
Hypotension	<90	And	<60
Normotension	<120	And	<80
Prehypertension	120–129	And	<80
Hypertension Stage 1	130–139	Or	80–89
Hypertension Stage 2	140–180	Or	90–120

**Table 2:** Classification accuracy performance with 95% confidence interval and *p*-value for each input signal.

Input	Number of Convolutional Layers	Train Accuracy	Validation Accuracy	Test Accuracy	*p*-Value
PPG	1 layer	47.70–49.10%	47.51–49.29%	47.94–49.66%	$5.55\times 10^{-8}$
	2 layers	49.45–50.55%	49.45–50.15%	49.45–50.15%	
	3 layers	53.56–57.64%	53.58–58.42%	53.43–56.57%	
	4 layers	54.39–60.42%	54.39–60.42%	53.82–58.58%	
	5 layers	59.50–62.90%	59.63–63.57%	57.97–61.23%	
ECG	1 layer	68.89–71.11%	68.89–71.10%	63.44–69.36%	$1.25\times 10^{-5}$
	2 layers	71.94–73.66%	71.94–73.66%	69.64–71.96%	
	3 layers	69.33–73.07%	69.62–73.98%	67.87–70.93%	
	4 layers	70.63–74.57%	70.71–75.29%	69.40–72.20%	
	5 layers	76.16–79.84%	76.16–79.84%	75.63–79.57%	
PPG and ECG	1 layer	92.34–94.06%	92.34–94.06%	88.76–86.04%	$4.72\times 10^{-12}$
	2 layers	99.45–99.82%	99.45–99.82%	93.55–94.23%	
	3 layers	100%	100%	94.05–94.64%	
	4 layers	100%	100%	94.37–94.97%	
	5 layers	100%	100%	94.56–95.15%	

**Table 3:** The performance results for classifying each blood pressure level of test data using the proposed concatenated CNN architecture.

CNN Architectures	Configuration (Parallel)	Precision	Recall	F1-Score	AUC
Architecture 1	1 layer (number of filters 8)	0.86–0.88	0.85–0.87	0.86–0.87	0.96–0.97
Architecture 2	2 layers (number of filters 8 and 16)	0.92–0.93	0.92–0.93	0.92–0.93	0.97–0.98
Architecture 3	3 layers (number of filters 8, 16, and 32)	0.93–0.94	0.93–0.94	0.93–0.94	0.98–0.99
Architecture 4	4 layers (number of filters 8, 16, 32, and 64)	0.93–0.94	0.93–0.94	0.93–0.94	0.98–0.99
Architecture 5	5 layers (number of filters 8, 16, 32, 64, and 128)	0.93–0.95	0.93–0.95	0.93–0.95	0.98–0.99

**Table 4:** The performance comparison with previous studies.

Authors	Dataset	No of Classes	Method	Performance
Wu et al. [31]	PPG signal	2 classes (normal and abnormal)	CWT and 2D CNN	Accuracy of 90%
Sun et al. [32]	PPG signal	3 classes (normotension, prehypertension, and hypertension)	HHT and 2D CNN	Accuracy of 93.54%
Yen et al. [33]	PPG signal	4 classes (normotension, prehypertension, hypertension stage 1, and hypertension stage 2)	BILSTM and ResNet	Accuracy of 76%
Soh et al. [42]	ECG signal	2 classes (normotension and hypertension)	CNN	Accuracy of 99.99%
Jain et al. [43]	ECG signal	2 classes (normotension and hypertension)	CNN	Accuracy of 99.68%
Rajput et al. [44]	ECG signal	2 classes (normotension and hypertension)	OWFB	Accuracy of 99.95%
Sharma et al. [45]	ECG signal	2 classes (normotension and hypertension)	SVM	Accuracy of 98.05%
Liang et al. [46]	PPG and ECG signal	3 classes (normotension, prehypertension, and hypertension)	K-NN	F1 score of 88.49%
Kuzmanov et al. [47]	PPG and ECG signal	2 classes (hypotension and not hypotension)	LSTM and CNN	Accuracy of 76%
Kuzmanov et al. [48]	PPG and ECG signal	3 classes (normotension, prehypertension, and hypertension)	GRU and CNN	Accuracy of 78%
Our study	PPG and ECG signal	5 classes (hypotension, normotension, prehypertension, hypertension stage 1, and hypertension stage 2)	Concatenated 1D CNN	Accuracy of 95%

## Data Availability

The dataset used in this study can be accessed via the Physionet website at https://physionet.org/content/mimiciii/1.4/ (accessed on 20 January 2022).
